# Ta_2_O_5_/rGO Nanocomposite Modified Electrodes for Detection of Tryptophan *through* Electrochemical Route

**DOI:** 10.3390/nano9060811

**Published:** 2019-05-28

**Authors:** Shun Zhou, Zefeng Deng, Zhongkang Wu, Mei Xie, Yaling Tian, Yiyong Wu, Jun Liu, Guangli Li, Quanguo He

**Affiliations:** Hunan Key Laboratory of Biomedical Nanomaterials and Devices, School of Life Science and Chemistry, Hunan University of Technology, Zhuzhou 412007, China; zs1187357805@163.com (S.Z.); dzf18073872860@163.com (Z.D.); wzk18897496745@163.com (Z.W.); meixie1015@126.com (M.X.); tianyaling0212@163.com (Y.T.); wyy5082010@163.com (Y.W.); guangli010@hut.edu.cn (G.L.)

**Keywords:** Ta_2_O_5_-rGO composite, tryptophan, electrochemical detection, second derivative linear scan voltammetry

## Abstract

l-tryptophan is one of the eight kinds of essential amino acids for sustainable human life activity. It is common to detect the concentration of tryptophan in human serum for diagnosing and preventing brain related diseases. Herein, in this study, GCE (glassy carbon electrode) modified by Ta_2_O_5_-reduced graphene oxide (-rGO) composite (Ta_2_O_5_-rGO-GCE) is synthesized by the hydrothermal synthesis-calcination methods, which is used for detecting the concentration of tryptophan in human serum under the as-obtained optimal detection conditions. As a result, the obtained Ta_2_O_5_-rGO-GCE shows larger electrochemical activity area than other bare GCE and rGO-GCE due to the synergistic effect of Ta_2_O_5_ NPs and rGO. Meanwhile, Ta_2_O_5_-rGO-GCE shows an excellent response to tryptophan during the oxidation process in 0.1 M phosphate buffer solution (pH = 6). Moreover, three wide linear detection range (1.0–8.0 μM, 8.0–80 μM and 80–800 μM) and a low limit of detection (LOD) of 0.84 μM (S/N = 3) in the detection of tryptophan are also presented, showing the larger linear ranges and lower detection limit by employing Ta_2_O_5_-rGO-GCE. Finally, the as-proposed Ta_2_O_5_-rGO-GCE with satisfactory recoveries (101~106%) is successfully realized for the detection of tryptophan in human serum. The synthesis of Ta_2_O_5_-rGO-GCE in this article could provide a slight view for the synthesis of other electrochemical catalytic systems in detection of trace substance in human serum.

## 1. Introduction

l-tryptophan is an important constituent of proteins, and it is also an indispensable component in human nutrition for building and keeping a positive nitrogen balance. More importantly, l-tryptophan is one of the eight essential amino acids for human’s normal daily activity, including brain functions and neuronal regulatory mechanisms [1,2]. Many reports find that serotonin and melatonin are related to tryptophan, and toxic metabolites are produced in the brain when improperly metabolized, which is considered to be one of possible reasons for schizophrenia. Therefore, detection of tryptophan for preventing brain diseases is more significant [3,4]. Recently, a variety of methods are used in the measurement of tryptophan, including liquid chromatography, gas chromatography-mass spectrometry, spectroscopic detection, etc. [5,6,7]. These methods, with reliable and effective properties, are widely used for biological analysis, such as the precise measurement of dopamine or tryptophan. Nevertheless, there are still some disadvantages in using these methods, such as long detection time, expensive equipment and complex analytic routes [8,9].

In recent years, electrochemical analysis is considered as a green, highly sensitive and low-cost method for the detection of small biological molecules compared with the above-mentioned methods. Additionally, because of the double-bond of indolyl, tryptophan is easily oxidized for forming a C–N double bond through the electrochemical route. Thus, electrochemical method is commonly used in the detection of tryptophan. For example, Yeon and co-workers prepared reduced graphene oxide (rGO) decorated with tin oxide (SnO_2_) nanoparticles, which was used in the modification of glassy carbon electrode (GCE) for enhancing the detection of tryptophan (Trp). The lower detection limit of Trp was identified at 0.04 μM (S/N = 3), and the linear relationship range was found to the Trp concentration of 1–100 μM. The sensor demonstrated an excellent selectivity, good stability, and reproducibility. It could be used for the detection of Trp in the milk and amino acid injection samples [10].

Among all nanomaterials, rGO, with good electroconductibility, is wildly used in electrochemical analysis. When rGO composited with a semiconductor, the catalytic performance could be improved because of the synergistic effect. The good catalytic performance of a semiconductor and the electroconductibility of rGO could improve the detection sensitivity of small biological molecules. Many kinds of semiconductors coupling with rGO, such as Cu_2_O, MnO_2_, Fe_3_O_4_, TiO_2_, etc., are carried out for electrochemical analytic applications [11,12,13,14,15]. These semiconductors possess high catalytic activities due to their special electronic structure and redox performance. Recently, Ta_2_O_5_ was found to be an excellent candidate for electrochemical sensors and biosensors [16,17]. Ta_2_O_5_ semiconductor, as a transition metal oxide, shows a wide band-gap (4.0 eV) and can be used as the catalyst for various applications [18,19]. For example, Gurung and co-workers prepared Ti/Ta_2_O_5_-SnO_2_ electrodes for the electrochemical oxidation (EO) of carbamazepine (CBZ) synthetic solutions and real membrane bioreactor (MBR) effluent. The EO based on the use of Ti/Ta_2_O_5_-SnO_2_ electrode with high catalytic performance was found to be a reliable method for removing CBZ from contaminated waters [20]. In addition, Ta_2_O_5_ has excellent chemical and thermal stabilities in practical application. Therefore, it is a potential candidate for the fabrication of the electrochemical sensors and biosensors. When the Ta_2_O_5_ is composited with other carbon materials, such as rGO and carbon nanotubes, the detection sensitivity and catalytic performance may be improved due to the synergistic catalytic effect between Ta_2_O_5_ and carbon materials.

In our best knowledge, fewer literatures have reported the synthesis of Ta_2_O_5_-rGO composite for the detection of tryptophan. Herein, in this paper, Ta_2_O_5_-rGO composite is synthesized for the sensitive detection of tryptophan. Ta_2_O_5_ nanoparticles are prepared by combining hydrothermal synthesis-calcination methods, and rGO is obtained by the modified Hummers’ method and the electrochemical reduction method. GCE modified with this composite structure is used in the electrochemical detection of tryptophan. A lot of parameters including solution pH, accumulation potential and accumulation time are also investigated. Finally, this electrode is employed for the detection of tryptophan in human serum samples.

## 2. Experimental Section

### 2.1. Materials and Chemicals

Tantalum chloride (TaCl_5_), diethanol amine (DEOA), potassium permanganate (KMnO_4_), graphite, sulfuric acid (H_2_SO_4_), hydrogen peroxide (H_2_O_2_), α-aluminium oxide (α-Al_2_O_3_, particle size: 1.0 μm, 0.3 μm and 0.05 μm), disodium phosphate dodecahydrate (Na_2_HPO_4_∙12H_2_O), sodium nitrate (NaNO_3_), sodium dihydrogen phosphate (NaH_2_PO_4_), potassium ferricyanide (K_3_Fe(CN)_6_), potassium ferrocyanide (K_4_Fe(CN)_6_) and ethyl alcohol were purchased from Sinopharm Chemical Reagent Co., Ltd. (Shanghai, China). l-tryptophan was purchased from Aladdin Bio-Chem Technology Co., Ltd. (Shanghai, China). Human serums were taken from people’s hospital of Zhuzhou. During these experimental process, ultrapure water (18.2 MΩ) was used generally. All of the reagents were utilized directly without any further purification.

### 2.2. Preparation of Ta_2_O_5_ Nanoparticles

**Scheme 1:**
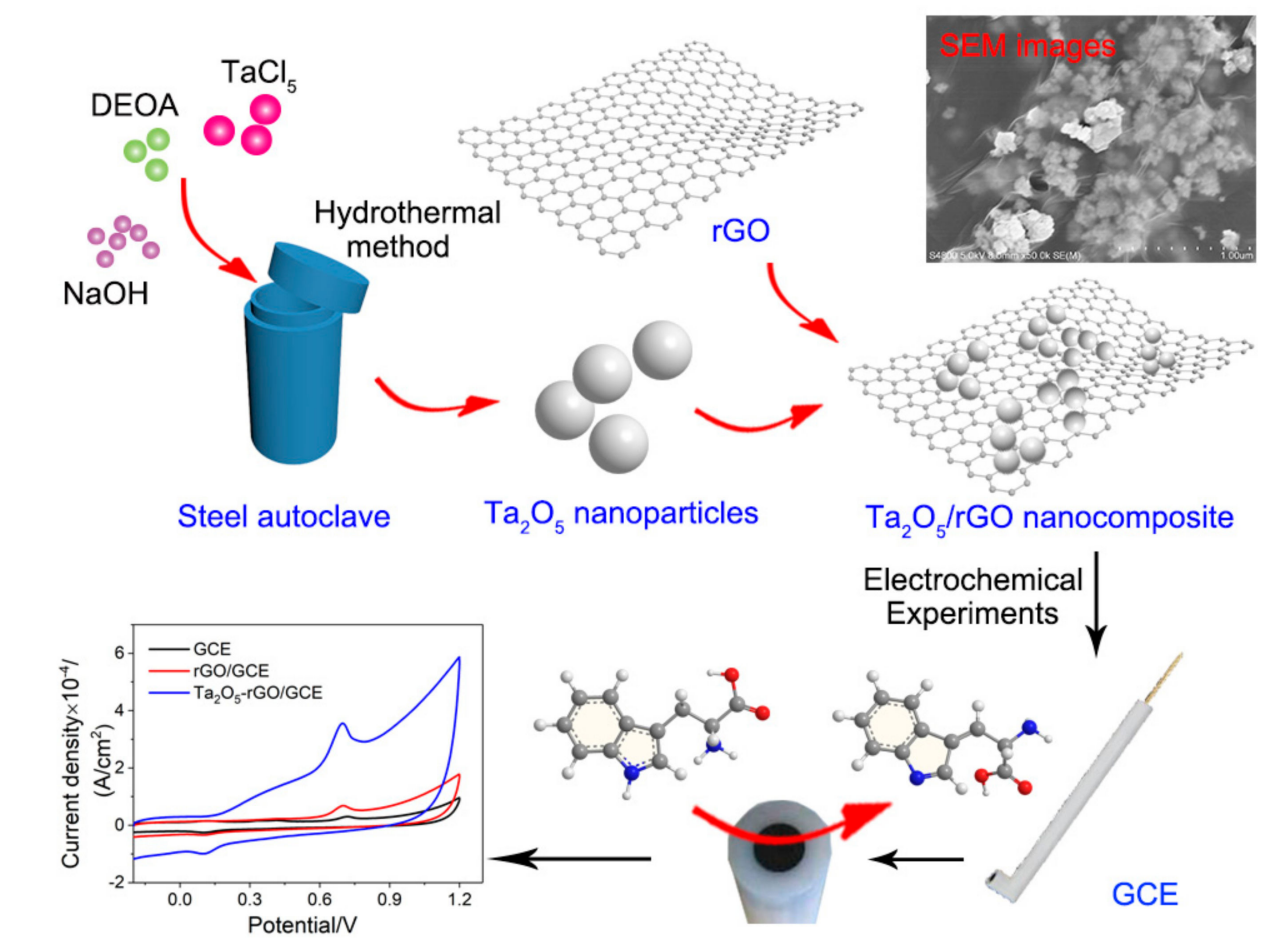
The synthesized and electrochemical processes of Ta_2_O_5_-rGO for the detection of tryptophan.

All of these synthetic processes and correspondingly electrochemical processes are illustrated in Scheme 1. Firstly, the Ta_2_O_5_ nanoparticles were prepared by hydrothermal synthesis-calcination method [21]. Typically, 0.05 mL of diethanol amine was added into 15 mL of TaCl_5_ solution (0.05 M) as a stabilizer. Then, 5 mL of NaOH solution (0.01 M) was subsequently added, and stirred for 1 h at room temperature. Afterwards, this solution was added into 100 mL of stainless-steel autoclave with Teflon-lined. The sealed autoclave was heated at 80 °C for 48 h. The precipitate was washed by deionized water and ethanol for three times after cooling into room temperature. Then, as-obtained sample was dried in vacuum oven at room temperature for 12 h. Finally, the dried Ta_2_O_5_ powder was calcined at 700 °C for 3 h.

### 2.3. Synthesis of Ta_2_O_5_/GO Composites

In this experiment, modified Hummers’ method was employed for preparing graphene oxide (GO), which is reported in our previous report [22]. Typically, 0.5 g of graphite powder and 0.5 g of NaNO_3_ were slowly added into concentrated H_2_SO_4_ (98 wt. %, 23 mL, cooled to 0 °C) under mechanical stirring. Then, keeping the whole temperature lower than 5 °C, 3.0 g of KMnO_4_ was added slowly into the above solution. A mash formed after the temperature raised to 35 °C, which was kept under stirring for 2 h. Subsequently, 40 mL of water was slowly added into the above mash under the temperature lower than 50 °C. After finishing the water adding, the temperature raised to 95 °C for 0.5 h. The above solution was added into 20 mL of 30% H_2_O_2_ in batches after adding 100 mL of water. A brown suspension was obtained by adding 150 mL of hydrochloric acid (1:10). Afterwards, the product was washed with 150 mL of H_2_O and collected by the suction filter. The final product was dried in vacuum oven at 50 °C for 12 h. Finally, 100 mL of GO solution (1 mg GO/mL water) was prepared for further using. Ta_2_O_5_-GO nanocomposites were obtained by adding 20 mg of Ta_2_O_5_ NPs into 20 mL of GO solution under ultrasound for 2 h.

### 2.4. Fabrication of Ta_2_O_5_-GO-Modified GCE

Before loaded with Ta_2_O_5_-GO, the GCEs were polished by α-Al_2_O_3_ powder with different sizes (by using them with size of 1.0 μm, 0.3 μm and 0.05 μm in sequence). Then, the GCEs were washed by ethyl alcohol and water under ultrasound for 1 min. 5 μL of Ta_2_O_5_-GO/GCEs (1 mg/mL) were prepared by drop-casting of Ta_2_O_5_-GO suspension onto the GCEs, and drying under infrared lamp. For comparison, graphene oxide-modified GCEs (GO/GCE) were also prepared by the same method. Finally, the Ta_2_O_5_-RGO/GCE was obtained after the GO in Ta_2_O_5_-GO/GCE was reduced by electrochemical reduction method under the potential of −1.5 V for 120 s (pH = 6.0 phosphate buffer solution (PBS)).

### 2.5. Characterization

Powder X-ray diffraction (XRD) patterns were operated with an X-ray diffractometer (PANalytical, Amsterdam, Holland) operating at 40 kV and 40 mA with Cu Kα radiation (λ = 0.1542 nm). Scanning electron microscopy (SEM) images were taken on a Hitachi S4800 (Hitachi, Tokyo, Japan) operated at 5 kV. Transmission electron microscopy (TEM) images were carried out by JEOL JEM-2010 (HT, Tokyo, Japan), operated at 200 kV. The electrochemical behaviors of as-prepared samples were tested by electrochemical workstation (CHI760E, Shanghai Chenhua Instrument Co., Ltd., Shanghai, China).

### 2.6. Electrochemical Experiments

All electrochemical experiments, including cyclic voltammetry (CV) and second-order derivative linear sweep voltammetry (SDLSV), were carried out by using bare or modified GCEs as work electrodes, platinum wire electrode as counter electrode, and saturated calomel electrode (SCE) as reference electrode. 1 × 10^−5^ mol/L of freshly-prepared tryptophan in 0.1 M of PBS were used to test the electrochemical response of CV on Ta_2_O_5_-rGO-GCE. SDLSV was used to measure the sensing performance of tartrazine on Ta_2_O_5_-rGO-GCE in an electrochemical cell containing 0.1 M of PBS. The scan rate is set as 100 mV/s in both CVs and SDLSV testing. Before staring the test process, a suitable accumulation period was carried out under stirring at 500 rpm. The potential scan ranges were −0.6–1.2 V for CV and 0.5–1.2 V for SDLSV.

### 2.7. Detection of Tryptophan in Human Serum

The detection of tryptophan in human serum is carried out by the standard addition method after the best detection conditions were obtained. Typically, 1 mL of human serum sample was diluted to 10 mL by 1.0 M of PBS (pH = 6.0) and ultrapure water. Moreover, another two solutions (10 mL) are prepared by the same method with further adding 1 mL and 2 mL of standard tryptophan solution (a certain concentration), respectively. Then the CV tests are carried out for detection of the concentration of tryptophan in human serum.

## 3. Results and Discussion

### 3.1. Structural and Morphologic Characterization of Ta_2_O_5_ and Ta_2_O_5_-GO

**Figure 1:**
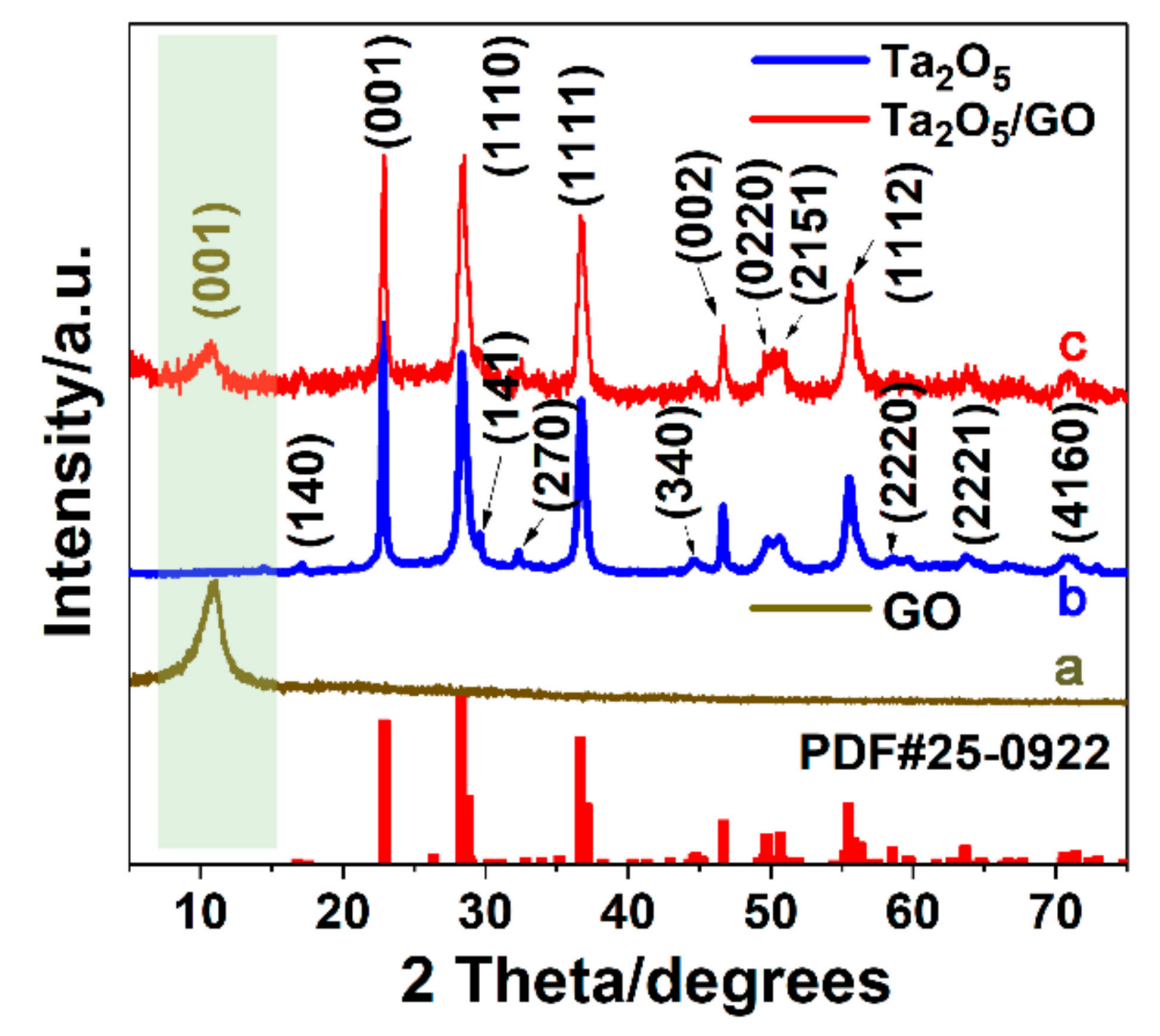
X-ray diffraction (XRD) patterns of graphene oxide (GO) nanosheets (**a**), pure Ta_2_O_5_ nanoparticles (**b**) and Ta_2_O_5_-GO composites (**c**).

The structures of GO nanosheets, pure Ta_2_O_5_ nanoparticles and Ta_2_O_5_-GO composites are characterized by XRD technique. As presented in Figure 1, only a strong diffraction peak at 10° is observed in curve a, which is attributed to the (001) plane of GO, indicating the GO is synthesized successfully. The sharp diffraction peaks are observed in curve b, indicating the high crystallinity of as-synthesized Ta_2_O_5_ nanoparticles. Moreover, the standard Joint Committee Powder Diffraction Standards (JCPDS) card of the pure orthorhombic Ta_2_O_5_ (25-0922, red columns) are used for comparison. The obvious diffraction peaks of Ta_2_O_5_ between 15° to 75° are in accordance with the standard JCPDS card, which are indexed to (140), (001), (1110), (141), (270), (1111), (340), (002), (0220), (2151), (1112), (2220), (2221) and (4160) planes of orthorhombic Ta_2_O_5_. Thus, the result indicates that the Ta_2_O_5_ nanoparticles are synthesized successfully. More importantly, the XRD pattern of Ta_2_O_5_-GO composites is presented in curve c. Both the (001) plane of GO and other planes of Ta_2_O_5_ are shown, meaning that the Ta_2_O_5_-GO composites are synthesized successfully.

**Figure 2:**
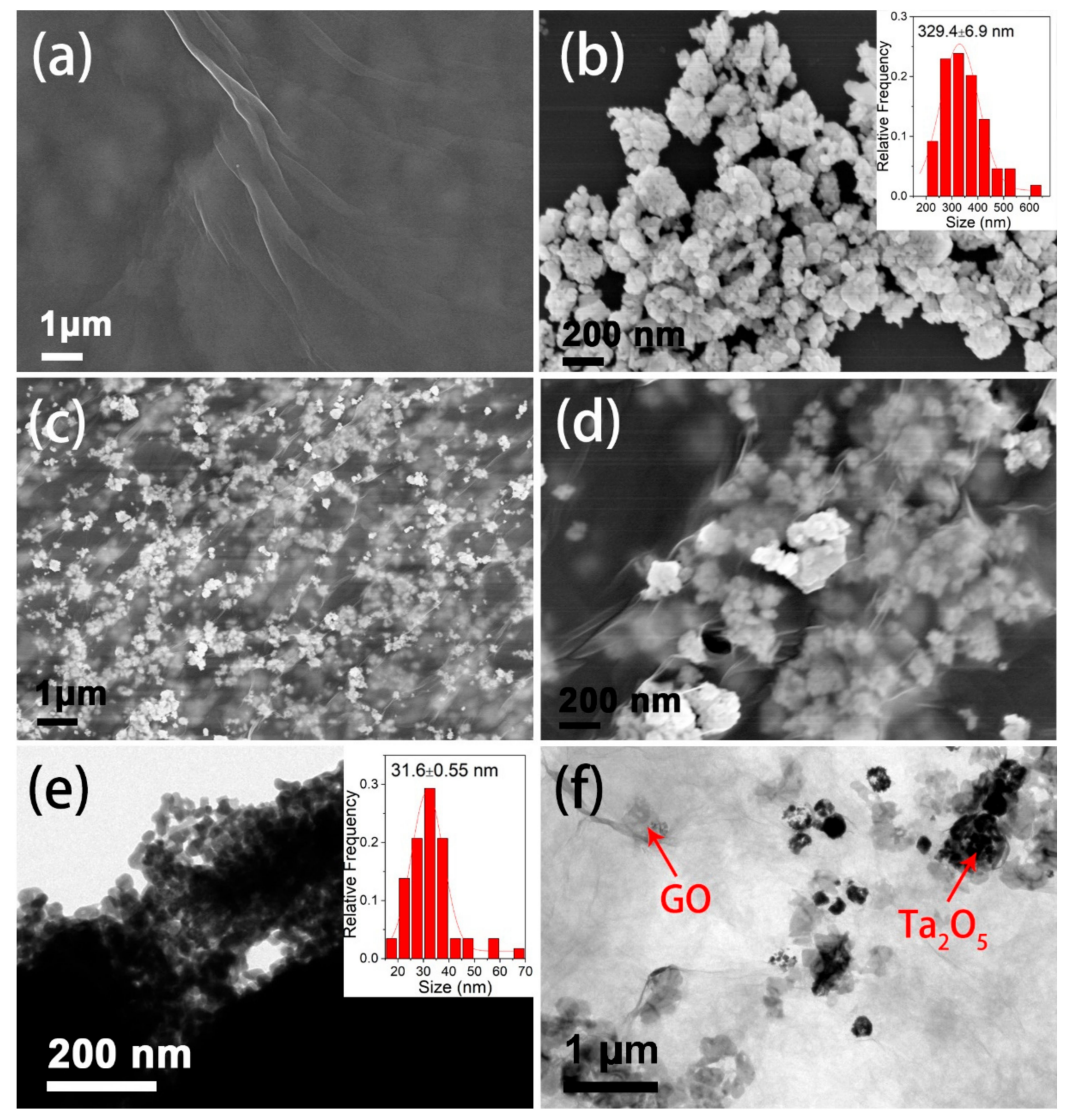
Scanninig electron microscopy (SEM) images of the pure GO (**a**), Ta_2_O_5_ nanoparticles with the inset of the size distribution (**b**) and Ta_2_O_5_-GO composite nanoparticles (**c**,**d**). Transmission electron microscopy (TEM) images of the as-prepared Ta_2_O_5_ nanoparticles with the inset of the size distribution (**e**) and Ta_2_O_5_-GO composite nanoparticles (**f**).

The morphologies of as-prepared GO nanosheets, Ta_2_O_5_ nanoparticles and Ta_2_O_5_-GO composite nanoparticles are characterized by SEM. The layer-like and plicate structure of GO nanosheets is observed (Figure 2a). The Ta_2_O_5_ nanoparticles with good dispersibility are shown in Figure 2b and the particle size is estimated as 329.4 ± 6.9 nm (inset of Figure 2b). The large particles are formed because of the aggregation of the small particles. The size of these small particles is smaller than 100 nm, but the exact size could not be estimated due to the low-resolution of the SEM images. The SEM images of Ta_2_O_5_-GO are presented in Figure 2c,d. After Ta_2_O_5_ particles are composited with GO nanosheets, the excellent dispersibility is shown compared with the pure Ta_2_O_5_ nanoparticles. Many nanoparticles are dispersed on the surface of the 2D layer-structure GO. As shown in the amplifying SEM image (Figure 2d), the plicate layer of GO becomes more evident, and most of these Ta_2_O_5_ nanoparticles are coated on these GO layers, due to electron bombardment under high voltage propelling the electron transmission [23,24]. Moreover, the TEM images of Ta_2_O_5_ nanoparticles and Ta_2_O_5_-GO composite nanoparticles are also investigated. As shown in Figure 2e, the smaller Ta_2_O_5_ nanoparticles with the size of 31.6 ± 0.55 nm (inset of Figure 2e) are aggregated to form the larger nanoparticles, which is in accordance with the SEM image (Figure 2b). The TEM image of Ta_2_O_5_-GO composite nanoparticles also shows that the GO nanosheets are coated with Ta_2_O_5_ nanoparticles, and the smaller Ta_2_O_5_ nanoparticles are dispersed well on the surface of GO nanosheets (Figure 2f). These results confirm that the Ta_2_O_5_-GO composite nanoparticles are obtained successfully.

### 3.2. Electrochemical Activity Area of Ta_2_O_5_-rGO-GCE Nanocomposites

**Figure 3:**
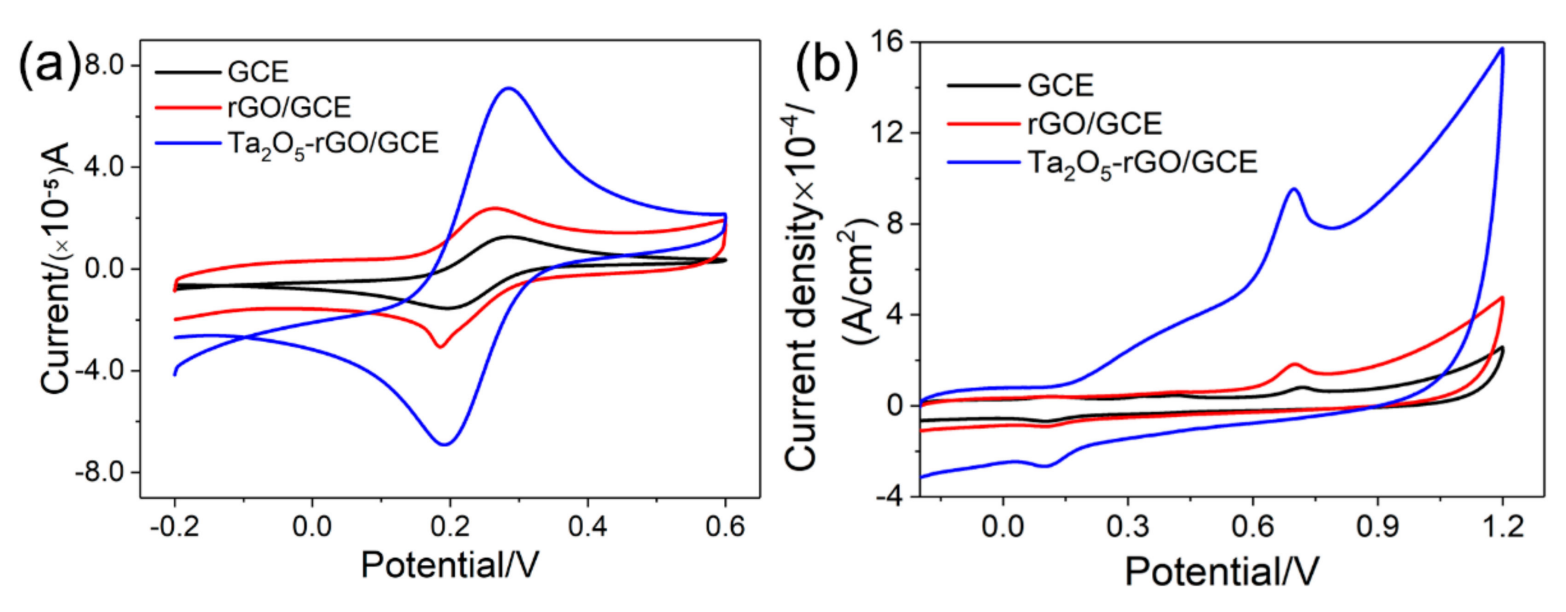
(**a**) Cyclic voltammetry (CV) curves of 5.0 × 10^−4^ mol/L of K_3_Fe(CN)_6_ at different electrodes (bare GCE, rGO-GCE and Ta_2_O_5_-rGO-GCE); (**b**) Electrochemical behaviours of tryptophan (1.0 × 10^−5^ mol/L) on bare GCE, rGO and Ta_2_O_5_-rGO electrodes (scan rate: 0.1 V/s, electrolyte: pH = 6.0, PBS).

The CV behaviors on bare GCE, rGO-GCE and Ta_2_O_5_-rGO-GCE in K_3_Fe(CN)_6_ solution are presented in Figure 3a. The intensity of oxidation peak currents (*i_pc_*) on GCE, rGO-GCE and Ta_2_O_5_-rGO-GCE is 1.560 × 10^−5^, 1.904 × 10^−5^ and 7.918 × 10^−5^ A, respectively. Therefore, according to Randles–Sevcik formula, the electrochemical activity areas could be calculated as:

*i_pc_* = 2.691 × 10^5^*n*^3/2^*D*^1/2^*ν*^1/2^*A C*. (1)

In this formula, *i_pc_* is reduction peak currents of K_3_Fe(CN)_6_, *n* is the transferred electron number during the redox reaction, *D* is diffusion coefficient of K_3_Fe(CN)_6_, *v* is scan rate (V/s), *A* is electrochemical activity area (cm^2^) and *C* is the concentration of K_3_Fe(CN)_6_ (mol/cm^3^). After the calculation, electrochemical activity area of the bare GCE is 0.047 cm^2^. On rGO-GCE, it is 0.057 cm^2^, a little larger than that of the bare GCE. However, the electrochemical activity area of the Ta_2_O_5_-rGO-GCE increases extensively to 0.239 cm^2^. After coated with rGO, the electrochemical performance of GCE is enhanced compared with the bare GCE, it probably because of the good conductibility of rGO. Moreover, the activity area of Ta_2_O_5_-rGO-GCE is larger than that of bare GCE and rGO-GCE, which indicates that the Ta_2_O_5_ modification could enhance the surface area of the bare electrode significantly. The enhancement of the electrochemical activity areas can not only enhance the efficiency for gathering of tryptophan on the modified electrodes, but also increase the catalytic sites of the modified electrodes, thus accelerating the redox reaction of tryptophan.

### 3.3. Electrochemical Behaviors of Tryptophan on Different Electrodes

The Electrochemical behaviors of tryptophan (1.0 × 10^−5^ mol/L) on the bare GCE, rGO-GCE and Ta_2_O_5_-rGO-GCE electrodes are shown in Figure 3b. The oxidation peak current of tryptophan on the bare GCE is 2.107 × 10^−6^ A, and the superficial area of GCE is 0.126 cm^2^. Therefore, the current density is 1.67 × 10^−5^ A/cm^2^. After GCE is coated with GO and under electrochemical reduction, the current of tryptophan on the rGO-GCE is improved to 5.707 × 10^−6^ A. Thus, the current density of rGO-GCE is 4.53 × 10^−5^ A/cm^2^ owing to the good electroconductibility of the rGO nanosheet. Moreover, the current of tryptophan on the Ta_2_O_5_-rGO-GCE is 1.742 × 10^−5^ A, and the current density is 1.38 × 10^−4^ A/cm^2^. It is about 3.05 times higher than that of rGO-GCE and 8.28 times higher than the bare GCE. Ta_2_O_5_ is a favorable catalyst, and the synergistic effect of Ta_2_O_5_ and rGO further promotes the increasing of peak current. Thus, for the detection of tryptophan, Ta_2_O_5_-rGO-GCE could improve the detection sensitivity.

### 3.4. Optimizations of Detection Conditions for Tryptophan

#### 3.4.1. Influence of the pH

**Figure 4:**
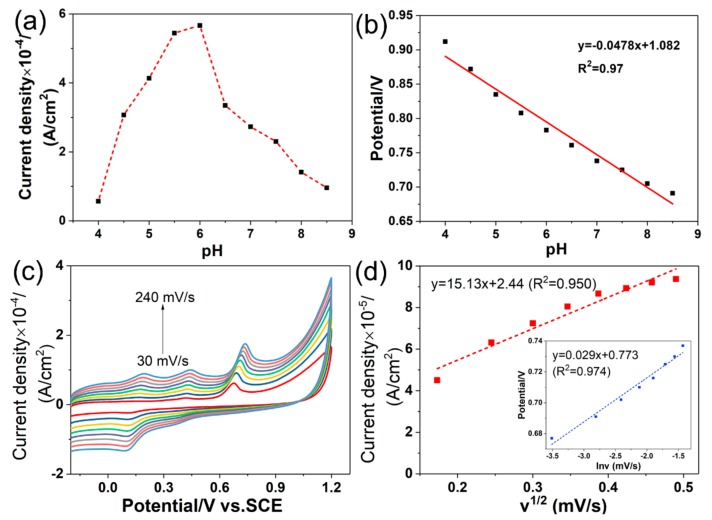
Effects of the pH on the oxidation peak currents of tryptophan (1.0 × 10^−5^ mol/L) at Ta_2_O_5_-rGO-GCE (**a**); and the linear relationship of the oxidation peak potential of tryptophan and the pH (**b**); the CV curves of tryptophan solution (1.0 × 10^−5^ mol/L) on Ta_2_O_5_-rGO-GCE under different scan rate (**c**); and the corresponding linear relationship of the oxidation peak current of tryptophan and the square root of scan rate, the inset is the relationship of the peak potential and the Napierian logarithm of the scan rate (**d**).

In Figure 4a, the CV response curves of tryptophan (1.0 × 10^−5^ mol/L) in PBS (0.1 mol/L) is presented at different pH values (4.0~8.5) (black line). With the increase of the pH, the oxidation peak current increases firstly and reduces later. The largest oxidation peak current is observed at pH = 6.0. Thus, the best pH value is proposed as 6.0 for the detection of tryptophan. Meanwhile, the excellent linear relationship of the oxidation peak potential and the pH is found (Figure 4b) with the linear equation of *E_p_*/V = −0.0478 pH + 1.082 (R^2^ = 0.97).

#### 3.4.2. Effect of the Scan Rate

**Figure 5:**
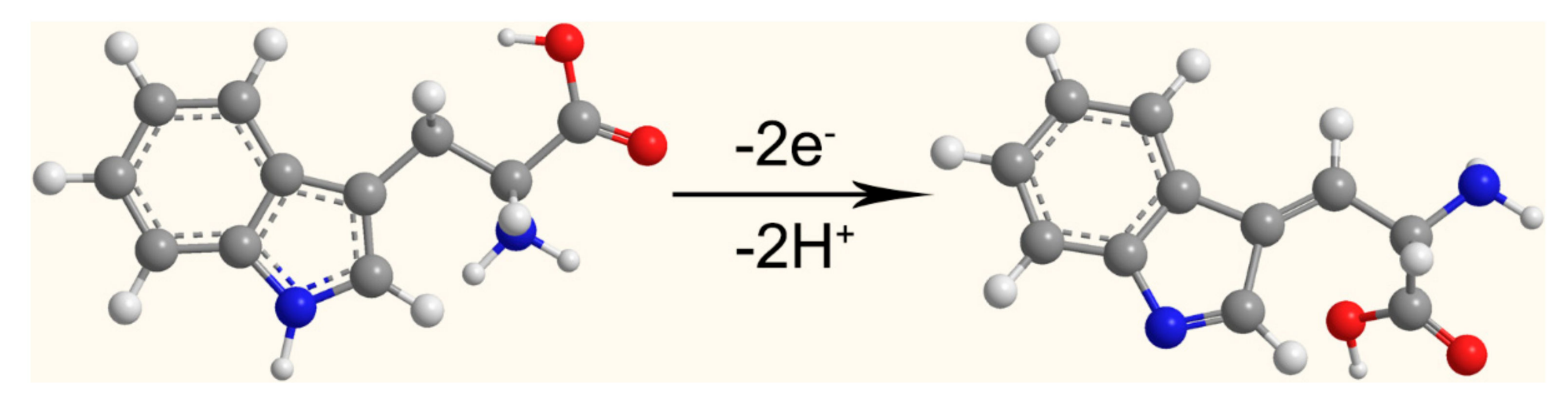
The oxidation pathway of tryptophan.

The CV curves of tryptophan (1.0 × 10^−5^ mol/L) on Ta_2_O_5_-rGO-GCE under different scan rate (30~240 mV/s) in PBS solution (0.1 mol/L, pH = 6.0) are presented in Figure 4c. The *i_pa_* of tryptophan increases gradually with the increase of the scan rate, but the background current also increases. At the same time, the linear relationship of *i_pa_* and the square root of the scan rate is observed in Figure 4d with the linear equation of *i_pa_* = 15.136*v*^1/2^ + 2.44 (R^2^ = 0.950). It identifies that the redox of tryptophan on Ta_2_O_5_-rGO-GCE is a diffusion-controlled process. The peak currents increase with the rising of the scan rate, but the background currents also improve correspondingly. Therefore, a suitable scan rate is chosen as 120 mV/s for improving the signal to noise ratio (SNR) and reducing the background current. As shown in the inset of Figure 4d, the oxidation peak potential (*E_pa_*) increases linearly with the Napierian logarithm of scan rate (ln*v*). The linear equation is *E_pa_* = 0.029 ln*v* + 0.773 (R^2^ = 0.974). Moreover, according to the following Lavrion equation:

*E_p_* = *E*^0^ + (*RT*/*αnF*) ln(*RTk*^0^/*αnF*) + (*RT*/*αnF*) ln*v,*

where *E*^0^ is standard potential (V), *T* is temperature (K), *α* is Electron transfer coefficient, *n* is electron transfer number, *k*^0^ is standard rate constant, *F* is Ferrari constant (*F* = 96.485 C/mol), *R* = 8.314 J/(K·mol) and *v* is scan rate. The inset of Figure 4d indicates that the slop (*RT*/*αnF*) is 0.029. As for an irreversible process, *α* is commonly assumed to be 0.5. Thus, *n* can be calculated as 1.77, which can be rounded to the nearest integer 2. It means that the oxidation of tryptophan on Ta_2_O_5_-rGO-GCE is an irreversible process containing two electrons and two protons, which is in accordance with the literature report [25]. The specific oxidation pathway of tryptophan is presented in Figure 5.

#### 3.4.3. Effect of the Accumulation Conditions

**Figure 6:**
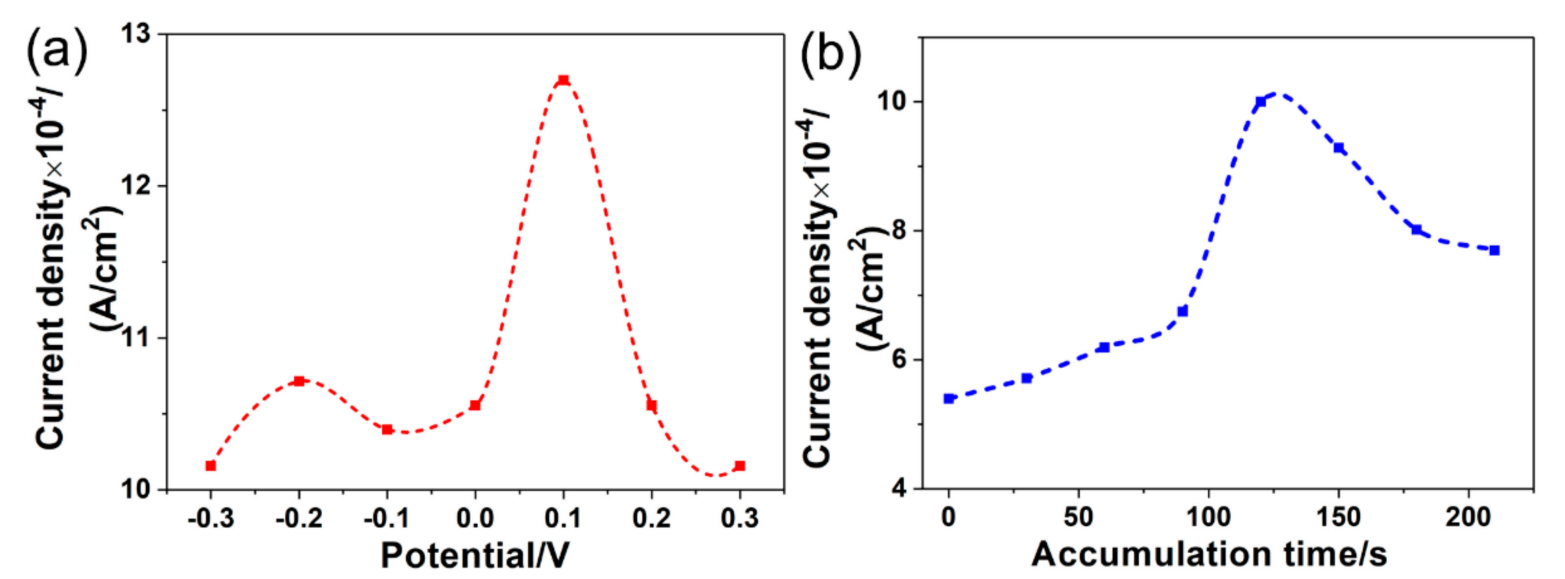
Effects of the accumulation potential (**a**) and the accumulation time (**b**) on the oxidation peak currents of 1.0 × 10^−5^ mol/L tryptophan at Ta_2_O_5_-rGO/GCE.

The accumulation way is used as a simple and useful method to improve the detection sensitivity. Thus, the accumulation potential and accumulation time for the oxidation current of tryptophan on Ta_2_O_5_-rGO-GCE are investigated in this section. Before testing the peak currents of tryptophan (1 × 10^−5^ mol/L), the accumulation process at different accumulation potentials (−0.3 to 0.3 V) for 120 s is carried out. The best accumulation potential is obtained at 0.1 V, which is presented in Figure 6a. Then, fixing the accumulation potential as 0.1 V, the accumulation time is investigated. Figure 6b shows the relationship between the accumulation time and the corresponding oxidation peak current. In the first 120 s, the oxidation peak currents increase rapidly. However, the oxidation peak currents decrease when the accumulation time further increases. Thus, in this study, 120 s is selected as the best accumulation time.

### 3.5. Stability of the Detection

**Figure 7:**
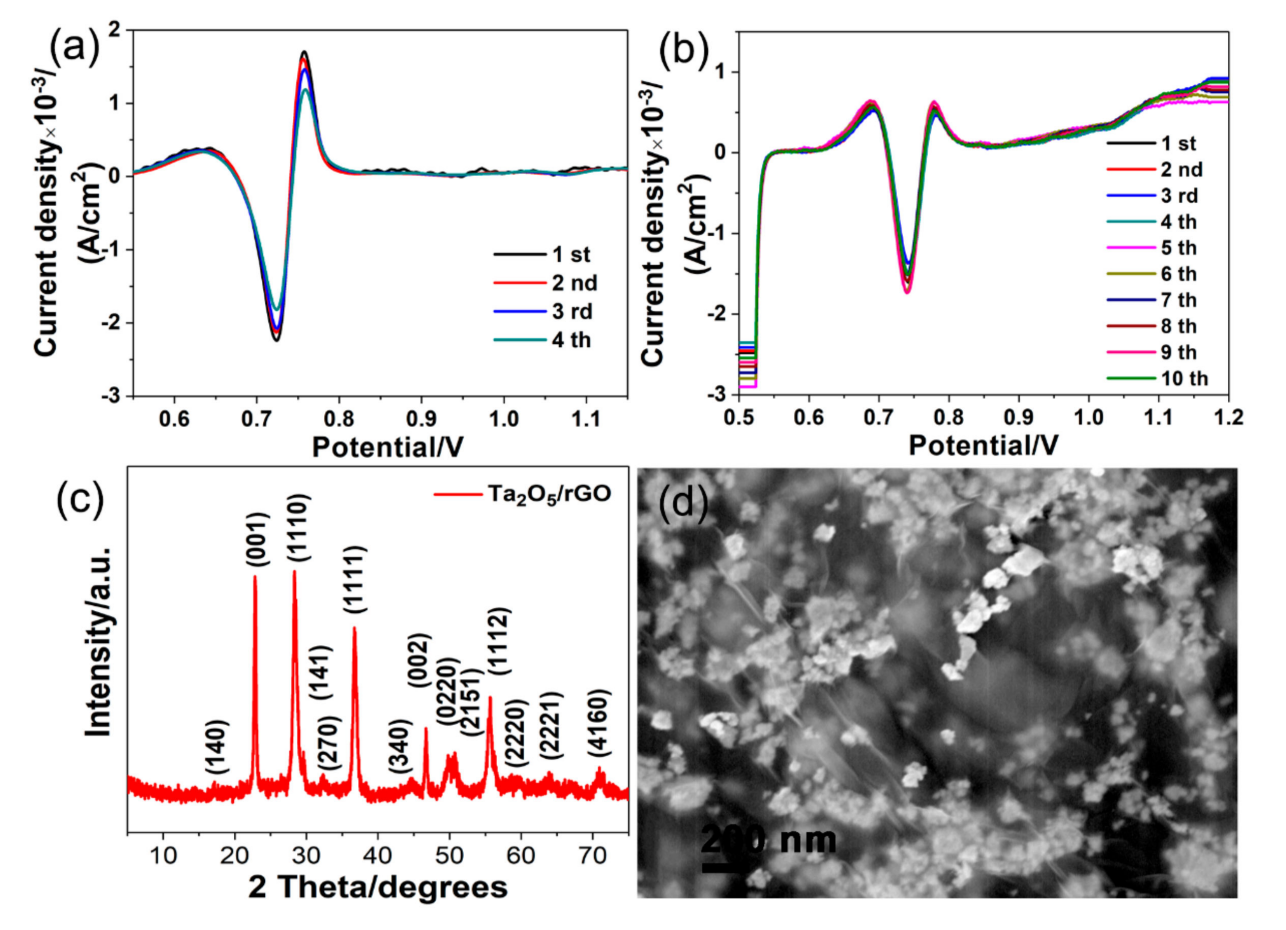
Second-order derivative linear scan voltammograms of tryptophan on four different Ta_2_O_5_-rGO-GCEs (**a**), on one Ta_2_O_5_-rGO-GCE repeated for 10 times (**b**); the XRD pattern (**c**) and the SEM image (**d**) of Ta_2_O_5_-rGO-GCE after ten-times repeated detection of tryptophan.

The stability of these electrodes in the detection of tryptophan is investigated for confirming the accuracy and practicability of the as-prepared Ta_2_O_5_-rGO-GCE before the detection of tryptophan in human serum. Under the best test condition, the reproducibility is examined by the detection of tryptophan (1 × 10^−5^ mol/L) on four different Ta_2_O_5_-rGO-GCEs by second-order derivative linear scan voltammograms (SDLSV) (Figure 7a). The relative standard deviation (RSD) is 8.629% (*n* = 4), and it suggest that the electrode fabrication is highly reproducible. Furthermore, ten-times repeated detection of tryptophan (1 × 10^−5^ mol/L) are also carried out in one electrode by SDLSV for checking the repeatability of Ta_2_O_5_-rGO-GCE (Figure 7b). The Ta_2_O_5_-rGO-GCE presents a good repeatability with the RSD of 8.625% (*n* = 10). Moreover, further structure and morphology characterization of Ta_2_O_5_-rGO-GCE after ten-times repeated detection are presented in Figure 7c,d. As shown in XRD pattern, the diffraction peak in 10° attributed to GO disappears, because of the reduction of GO to rGO. A broad peak of ~24° could be indexed to rGO, but it is submerged by the strong diffraction peaks of Ta_2_O_5_ nanoparticles. The SEM image (Figure 7d) shows that the obvious rGO sheets are coated with many Ta_2_O_5_ nanoparticles. These results indicate that the Ta_2_O_5_-rGO composite is stable after ten-times repeated detection.

### 3.6. Linear Ranges and Detection Limit

**Figure 8:**
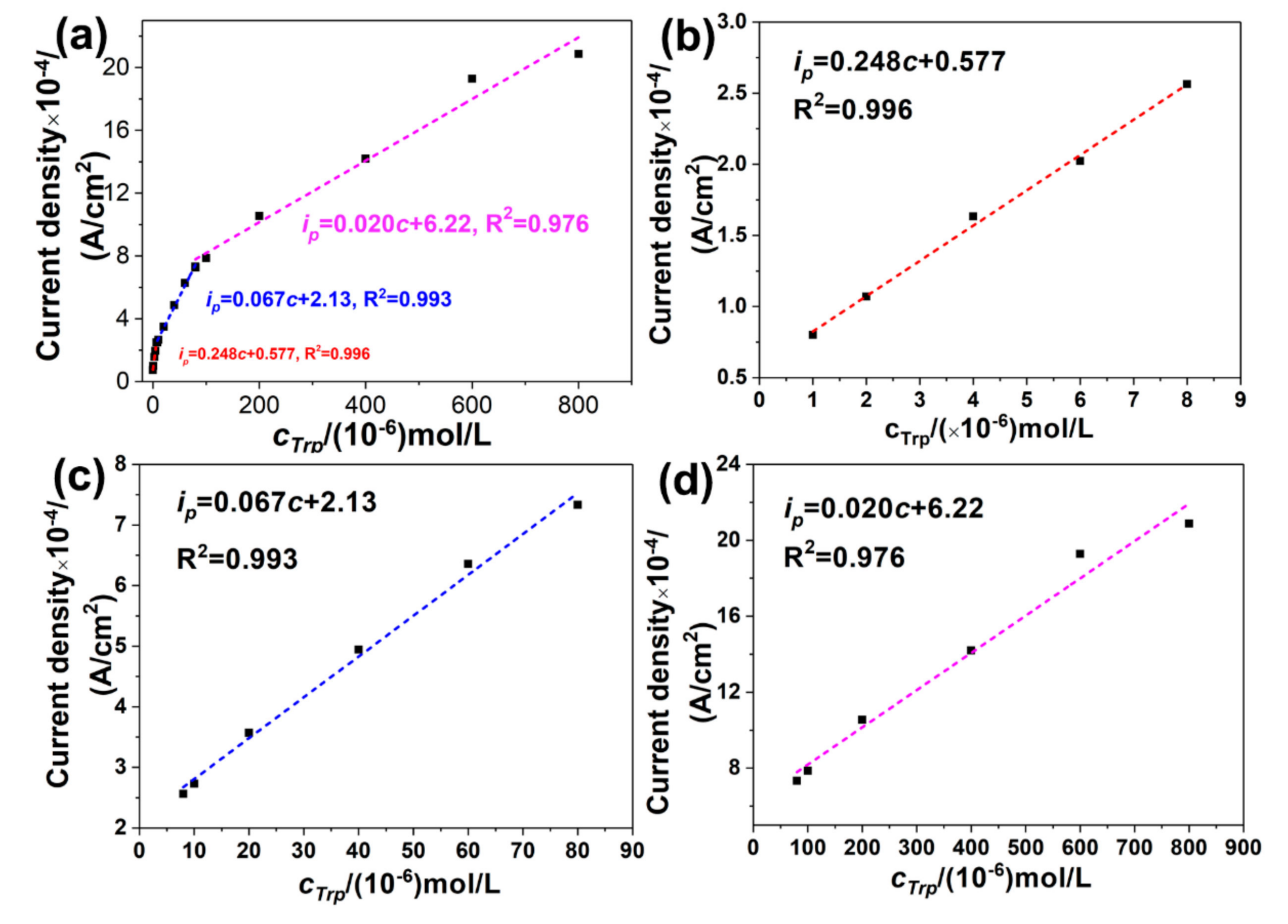
Three linear relationship of tryptophan in the range of 1.0 × 10^−6^~8.0 × 10^−4^ mol/L (**a**), the linear relationship of tryptophan in the range of 1 × 10^−6^ mol/L~8 × 10^−6^ mol/L (**b**), 8 × 10^−6^ mol/L~8 × 10^−5^ mol/L (**c**), and 8 × 10^−5^ mol/L~8 × 10^−4^ mol/L (**d**).

The quantitative analysis of tryptophan is investigated under the best detection conditions, and the concentrations of tryptophan are used in the range of 1.0 × 10^−6^~8.0 × 10^−4^ mol/L. Three good linear relationships of tryptophan are observed (Figure 8a). The first line is found at the range of 1 × 10^−6^ mol/L~8 × 10^−6^ mol/L, and the linear equation is *i_pa_* = 0.248*c* + 0.577 (R^2^ = 0.996) (Figure 8b). The second linear equation is *i_pa_* = 0.067*c* + 2.13 (R^2^ = 0.993) at the range of 8 × 10^−6^ mol/L~8 × 10^−5^ mol/L (Figure 8c). The last linear range is 8 × 10^−5^ mol/L~8 × 10^−4^ mol/L (Figure 8d), and the linear equation is *i_pa_* = 0.020*c* + 6.22 (R^2^ = 0.976). Thus, a good sensitivity of Ta_2_O_5_-rGO/GCE can be obtained in linear range of 1.0 × 10^−6^~8.0 × 10^−4^ mol/L. And the limit of detection (LOD) (S/N = 3) is estimated as 8.4 × 10^−7^ mol/L.

### 3.7. Practical Sample Detections

As an electrochemical technique, SDLSV are used extensively for the trace detection because of the high sensitivity and resolution. Therefore, in this section, the serum samples are measured by SDLSV under the best conditions. As presented in Table 1, the concentration of tryptophan in two human serum samples is 36.6 ± 10 μmol/L and 56.3 ± 10 μmol/L, with RSD of 1.34% (*n* = 3) and 2.82% (*n* = 3) respectively. As literature reported, the normal concentration of tryptophan in human serum is 40.05 ± 10.8 μmol/L [26]. These detected values include the normal concentration range. Moreover, the standard addition method is carried out for testing the recovery rate. Good recoveries (101~106%) show that the proposed Ta_2_O_5_-rGO-GCE has great application prospect in the detection of tryptophan in various real samples.

## 4. Conclusions

Herein, this paper presents a novel Ta_2_O_5_-rGO composite nanostructure for the modification of GCE. The Ta_2_O_5_ NPs are prepared by the hydrothermal synthesis-calcination method. The rGO nanosheets are synthesized by the modified Hummers’ method and electrochemical reduction. This Ta_2_O_5_-rGO-GCEs are applied for in vitro detections of tryptophan in human serum samples. As the result, the current density of Ta_2_O_5_-rGO-GCEs is larger than that of pure GCEs and rGO-GCEs due to the synergistic catalytic effect. This electrode shows high repeatability and reproducibility, meaning that it is useful in practical detection. More importantly, a wide linear range (from 1.0 μM to 800 μM) and a relative lower LODof 0.84 μM (S/N = 3) are also presented, which means this electrode could be applied in trace substance detection. Finally, the proposed Ta_2_O_5_-rGO-GCEs successfully realize the detection of tryptophan in human serum with the satisfactory recoveries (101~106%). It is a novel system in the detection of tryptophan in human serum samples, which can be a potential candidate for the detection of tryptophan in various actual samples.

## Figures and Tables

**Table 1:** The detection of Trp in human serum samples by second-order derivative linear sweep voltammetry (SDLSV) (*n* = 3).

Sample	Detected Amount (μmol/L)	RSD%	Added Amount (μmol/L)	Total Found Amount (μmol/L)	RSD%	Recovery
1#	36.6	1.34	20	56.7	1.6	101%
2#	56.3	2.82	40	99	1.08	106%
